# Precise Insertion of AttB Sequences in Goat Genome Using Enhanced Prime Editor

**DOI:** 10.3390/ijms25179486

**Published:** 2024-08-31

**Authors:** Aicong Li, Zhenliang Zhu, Jing Yang, Yayi Liu, Yong Zhang, Jun Liu

**Affiliations:** 1 Key Laboratory of Livestock Biology, Northwest A&F University, Yangling 712100, China; liaicong@nwafu.edu.cn (A.L.); zhuzhenliang@nwafu.edu.cn (Z.Z.); yangjing0129@nwafu.edu.cn (J.Y.); liuyayi586@nwafu.edu.cn (Y.L.); 2 College of Veterinary Medicine, Northwest A&F University, Yangling 712100, China

**Keywords:** prime editing, RNA motif, *goat*, *Rosa26*, *CCR5*

## Abstract

Prime editor, an editing tool based on the CRISPR/Cas9 system, allows for all 12 types of nucleotide exchanges and arbitrary indels in genomic sequences without the need for inducing DNA double-strand breaks. Despite its flexibility and precision, prime editing efficiency is still low and hindered by various factors such as target sites, editing types, and the length of the primer binding site. In this study, we developed a prime editing system by incorporating an RNA motif at the 3′ terminal of the pegRNA and integrating all twin prime editor factors into a single plasmid. These two strategies enhanced prime editing efficiency at target sites by up to 3.58-fold and 2.19-fold, respectively. Subsequently, enhanced prime editor was employed in *goat* cells and embryos to efficiently insert a 38 bp attB sequence into the *Gt(ROSA)26Sor* (*Rosa26*) and *C-C motif chemokine receptor 5* (*CCR5*) loci. The enhanced prime editor can mediate 11.9% and 6.8% editing efficiency in parthenogenetic activation of embryos through embryo microinjection. In summary, our study introduces a modified prime editing system with improved editing and transfection efficiency, making it more suitable for inserting foreign sequences into primary cells and embryos. These results broaden the potential applications of prime editing technologies in the production of transgenic animals.

## 1. Introduction

The CRISPR/Cas9 gene editing tool allows for precise and efficient fragment insertion into the genome [1]. In this system, the RNA-guided Cas9 nuclease causes double-strand breaks (DSBs), which can be repaired by non-homologous end joining (NHEJ), resulting in random small deletions or insertions (indels) or homology-directed repair (HDR), enabling the introduction of foreign genes carried by donor DNA [2]. CRISPR/Cas9, followed by HDR, provides a versatile method for generating transgenic animals when combined with somatic cell nuclear transfer (SCNT) and embryo transfer [3,4,5,6,7,8,9,10]. However, the limited efficiency of HDR, which is restricted to the S and G2 phases of the cell cycle, remains a significant obstacle [11]. The higher cost and technological complexity of SCNT have also increased the difficulty of the production of transgenic animals. Therefore, we expect to find a method of using microinjection to insert large fragments of exogenous genes into *goat* embryos.

Prime editing (PE), a novel approach derived from the CRISPR/Cas9 system, involves a nickase Cas9 (H840A), a Moloney murine leukemia virus reverse transcriptase (M-MLV RT), and a pegRNA containing a spacer sequence, a scaffold, a reverse transcriptase template (RTT), and a prime binding site (PBS) sequence [12]. PE enables base substitutions, small insertions, and deletions without the need for DSBs or donor DNA [13]. Compared to CRISPR/Cas9-HDR and base editing (BE), PE has lower off-target effects, increased safety, and enhanced flexibility while being unaffected by the cell cycle stage [14]. Large fragments of foreign genes can be integrated into the attB sequence inserted into the embryo genome by the twinPE system by site-specific recombination and can meet the needs of the production of transgenic animals. However, the efficiency of PE is still low. Consequently, provided that its efficiency can be improved, PE has the potential to achieve greater efficacy for precise gene editing by embryo microinjection.

Previously, multiple approaches have aimed at improving PE efficiency [15,16,17,18,19,20,21]. In this study, we improved the prime editor using the *HOXB13*-5′ UTR RNA motif and the integrated twinPE plasmid and demonstrated the incorporation of base substitutions, small insertions, and deletions, as well as the attB sequence at various target loci in HEK293T cells. Additionally, the attB sequence was introduced into the *Rosa26* and *CCR5* gene loci in *goat* cells and embryos with the aim of enabling the insertion of foreign genes into this attB site via site-specific recombination (SSR).

## 2. Results

### 2.1. The Addition of HOXB13-5′ UTR RNA Motif Enhances PE System Efficiency

The instability of the PBS and RTT in the pegRNA 3′ terminal region may lead to degradation [22,23] and potentially form an RNA double strand in the complementary PBS and spacer region [24]. Thus, the presence of an RNA motif capable of forming a stem-loop structure at the 3′ terminal region of pegRNA could potentially mitigate these challenges. Thus, we hypothesized that the integration of stabilizing RNA motifs at the pegRNA 3′ terminal region could enhance its functionality.

On the other hand, eukaryotic cells employ highly structured mRNA 5′ UTR regions to regulate mRNA translation initiation [25], as the stability of the mRNA 5′ UTR secondary structure impacts the recruitment and scanning process of ribosomes during translation initiation [26]. Thus, we hypothesized that the addition of a 5′ UTR RNA motif to the 3′ end of pegRNA could enhance its stability and functionality.

**Figure 1:**
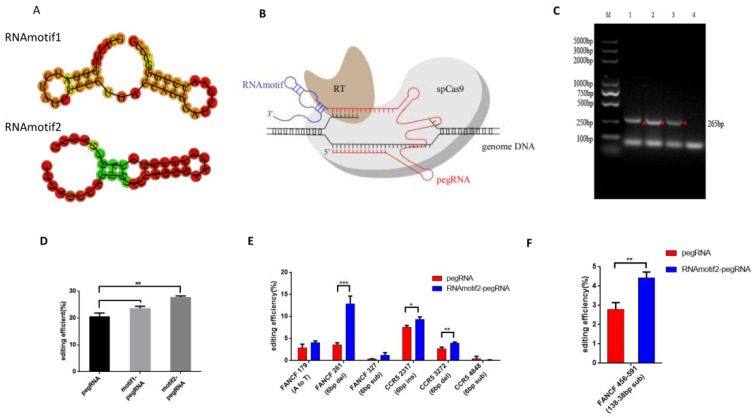
*HOXB13*-5′ UTR RNAmotif addition improves the efficiency of PE. (**A**) The schematic secondary structure of *HOXB13*-5′ UTR RNAmotif1 and RNAmotif2. (**B**) The schematic diagram of RNAmotif2-pegRNA. (**C**) BamHІ sequence insertion at *FANCF* 594 (6 bp ins) by PE3 and RNAmotif–PE3 detected by junction PCR. The red arrow indicates the destination strip. (**D**) Comparison of the prime editing efficiencies of PE3, RNAmotif1-PE3, and RNAmotif2-PE3 at *FANCF* 594 (6 bp ins). (**E**) Comparison of the prime editing efficiencies of PE3 and RNAmotif2-PE3 at target sites of *FANCF* and *CCR5*. (**F**) Comparison of the prime editing efficiencies of twinPE and RNAmotif2-twinPE at *FANCF* 456–591 (135–38bp sub). Data and error bars show the mean ± standard deviation (SEM) of three independent biological replicates (*n* = 3). *p*-values were obtained using *t*-tests. * *p* < 0.05, ** *p* < 0.01, *** *p* < 0.001.

First, we identified two sequences (RNAmotif1 and RNAmotif2) capable of forming stem-loop structures by utilizing the highly structured *HOXB13*-5′ UTR RNA motif (Figure 1A, Table 1). For this, we created an RNAmotif–PE system by incorporating these sequences into the 3′ end of pegRNA (Figure 1B). Subsequently, to induce a 6 bp insertion in *FA complementation group F*(*FANCF*), HEK293T cells were transfected with PE3, RNAmotif1-PE3, or RNAmotif2-PE3 constructs. Subsequent high-throughput DNA sequencing of genomic DNA samples indicated correct editing efficiencies of 20.64% (PE3), 23.5% (RNAmotif1-PE3), and 27.78% (RNAmotif2-PE3). Compared to WT pegRNA, these findings evidenced improved prime editing efficiencies at the specified target site of 1.14-fold (RNAmotif1-pegRNA) and 1.35-fold (RNAmotif2-pegRNA). Of note, RNAmotif2-pegRNA exhibited a more pronounced increase in efficiency overall (Figure 1C,D).

Next, we further analyzed the performance of RNAmotif2-pegRNA to mediate base substitution, 6 bp insertion, 6 bp deletions, and 6 bp substitutions at various gene loci and target sites. To verify whether RNAmotif2 has the same effect in the twinPE system, we used RNAmotif2-TwinPE to replace 135 bp to 38 bp at the *FANCF* site. Compared to WT pegRNA, RNAmotif2-pegRNA resulted in improved prime editing efficiencies at specific targets ranging between 1.41-fold and 3.58-fold for *FANCF* mutations and 1.23-fold and 1.46-fold for *CCR5* mutations. Of note, in four out of six experimental groups, statistically significant improvements were observed. Overall, these results indicated that pegRNAs linked with *HOXB13*-5′ UTR RNA motif2 resulted in higher prime editing efficiencies, albeit to differing extents (Figure 1E,F). The pegRNA sequences and primers used are shown in Table 2, Table 3 and Table 4.

### 2.2. The Integrated TwinPE System Improves the Transfection Efficiency of the TwinPE System

To initiate an editing process, multiple vectors have to be delivered into a single cell. The PE3 system consists of the PE2, pegRNA, and nsgRNA vectors, whereas the twinPE system consists of the PE2, pegRNA1, and pegRNA2 vectors. In contrast, to achieve site-specific recombination, the twinPE system requires the PE2, pegRNA1, pegRNA2, Bxb1, and donor vectors [13]. Low transfection efficiencies, particularly in difficult-to-transfect cell types, may result in an underestimation of PE efficiency due to impaired selection of transfected cells through antibiotic screening or flow cytometry. To overcome these obstacles and enhance the observed editing efficiency, we designed an integrated twinPE plasmid encompassing all twinPE components.

**Figure 2:**
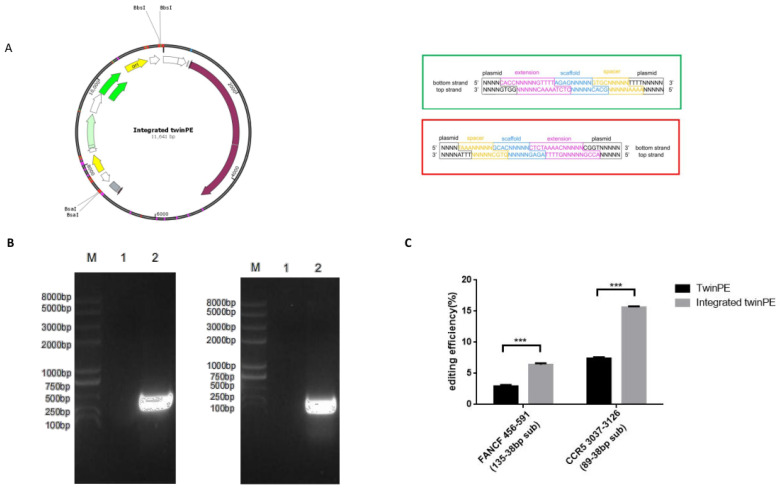
Integrated twinPE improves PE efficiency. (**A**) Integrated twinPE expression plasmid map and schematic diagram of the connection of each fragment of the integrated twinPE expression plasmid. (**B**) AttB sequence insertion at *FANCF* and *CCR5* by twinPE detected by junction PCR. (**C**) Comparison of the prime editing efficiencies of twinPE and integrated twinPE. Data and error bars show the mean ± standard deviation (SEM) of three independent biological replicates (*n* = 3). *p*-values were obtained using *t*-tests. *** *p* < 0.001.

The integrated twinPE plasmid contains two BbsІ and two BsaІ restriction sites, allowing simultaneous insertion of the respective pegRNAs. First, we digested the integrated twinPE plasmid with BsaІ-HF^®^v2 (NEB) and BbsІ-HF^®^v2 (NEB) and isolated the plasmid fragments of 4.1 kb and 7.4 kb (Table 5). Then, the top and bottom oligonucleotides of pegRNA1 and pegRNA2 flanked by 5′ and 3′ indicated overhangs and 5′ phosphorylated were synthesized and annealed (Table 6). Finally, the isolated 4.1 kb and 7.4 kb fragments and pegRNA1 and pegRNA2 oligonucleotides were ligated to form the twinPE integrated plasmid specifically designed for the specified targeting site (Figure 2A).

Subsequently, we targeted *FANCF* for a 135 bp to 38 bp substitution and *CCR5* for an 89 bp to 38 bp substitution by transfecting HEK293T cells with the twinPE system and the integrated twinPE plasmid. Subsequent high-throughput DNA sequencing of genomic DNA samples indicated correct editing efficiencies at *FANCF* of 2.92% for twinPE and 6.4% for integrated twinPE. In addition, correct editing efficiencies at *CCR5* of 7.41% for twinPE and 15.63% for integrated twinPE were observed. Compared to the twinPE system, these findings suggested that the integrated twinPE system results in 2.15-fold higher prime editing efficiency (Figure 2B,C).

### 2.3. Enhanced Prime Editing in Goat Fibroblast Cells

Although PE has been successfully applied in various animal species, its efficiency is lower in non-model mammalian primary cells compared to cell lines [27,28,29,30,31,32]. Thus, in order to assess the enhanced prime editing efficiency in *goat* fibroblast cells, we selected the safe harbor loci *Rosa26* and *CCR5*, which both contain six pairs of predicted target sites for twinPE.

**Figure 3:**
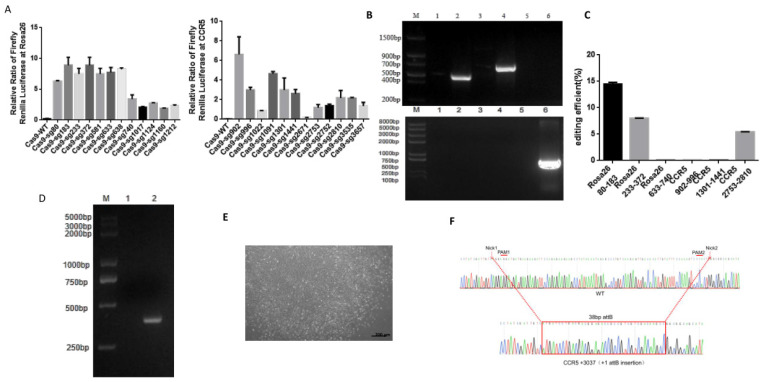
Insertion of attB sequence at *Rosa26* and *CCR5* gene loci of *goat* genome by twinPE. (**A**) Cutting efficiency of pegRNAs of *Rosa26* and *CCR5* detected by SSA. (**B**) AttB sequence insertion at *Rosa26* and *CCR5* of *goat* genome by twinPE detected by junction PCR. (**C**) Editing efficiency of inserting attB sequence at *Rosa26* and *CCR5* gene loci of *goat* genome by twinPE. (**D**) Monoclonal cells screened by limited dilution method (bar = 200 µm). (**E**) AttB sequence insertion at *Rosa26*–80–183 of monoclonal cells genome by twinPE can be detected by junction PCR. (**F**) Comparison of Sanger sequencing between WT *goat* genome amplification fragments and *Rosa26*–80–183 (attB ins) *goat* genome amplification fragments.

First, we evaluated the editing efficiency at these 12 pairs of target sites in HEK293T cells with the SSA reporter system. The SSA reporter system, consisting of pSSA-1-3, WT-PE2, sgRNA, and pRL-SV40, utilizes the WT-PE protein to induce a DSB at one of two firefly luciferase reporter genes within the pSSA-1-3 plasmid. Subsequently, following the expression of the intact firefly luciferase, its chemiluminescence value serves as a measure of the efficiency of pegRNAs’ cleavage activity [33]. Based on the SSA results, three pairs of target sites were selected for each gene locus, including *CCR5*–902–996, *CCR5*–1301–1441, *CCR5*–2752–2810, *Rosa26*–80–173, *Rosa26*–233–372, and *Rosa26*–633–740 (Figure 3A, Table 7).

To target specific genomic loci, we inserted a 38 bp attB sequence in the improved twinPEs and transfected *goat* fibroblast cells by electroporation. Following flow cytometry-mediated identification of positive cells, subsequent junction PCR and targeted deep sequencing demonstrated successful edits at two out of three *Rosa26* sites and one out of three *CCR5* sites. Of note, the prime editing efficiency at the *Rosa26*–80–173 locus peaked at 14.48% (Figure 3B,C). The primers for junction PCR and deep-targeted sequencing are shown in Table 8 and Table 9.

Finally, we selected the most efficient targeting site, *Rosa26*–80–173, for subsequent experiments. First, we screened monoclonal cells with the limited dilution method followed by junction PCR and Sanger sequencing (Figure 3D–F). Then, we screened out monoclonal cells that successfully inserted the attB sequence at the *Rosa26*–80–173 site in the *goat* genome as donor cells for nuclear transfer of somatic cells (SCNT) to produce transgenic *goats*.

### 2.4. Enhanced Prime Editing in Goat Embryos

**Figure 4:**
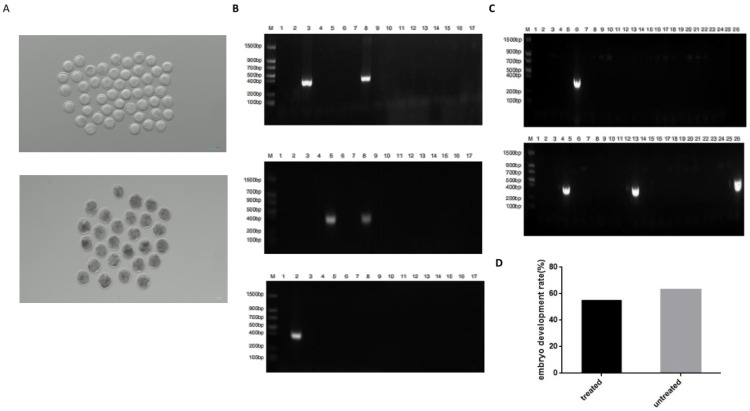
Insertion of the attB sequence at *Rosa26*–80–183 in the *goat* embryo genome by microinjection. (**A**) MII stage oocytes with regular morphology and uniform cytoplasm and embryos 5 days after microinjection. (**B**) Junction PCR identification of attB sequence insertion at *goat Rosa26*–80–183 of group 1 (**C**) Junction PCR identification of attB sequence insertion at *goat Rosa26*–80–183 of group 2. (**D**) Comparison of the embryo development rate of the treated groups (groups 1 and 2) and the untreated group (control group).

SCNT-mediated production of transgenic *goats* is a complex operation accompanied by high technical difficulty and costs. The transgenic *goat* production process can be accelerated through embryo microinjection by reducing the complexity and cost of the process. Thus, we wanted to analyze the feasibility of introducing attB sequences in *goat* embryos through twinPE microinjection. First, we generated the RNAmotif2-twinPE system targeting Rosa26–80–173 in vitro and microinjected 200 *goat* parthenogenetic activation embryos with 200 ng/µL PE2 mRNA, 50 ng/µL pegRNA1, and 50 ng/µL pegRNA2 (group 1) or 400 ng/µL PE2 mRNA, 100 ng/µL pegRNA1, and 100 ng/µL pegRNA2 (group 2). Then, 5 days following cultivation, we collected and cracked the surviving morulas and analyzed the twinPE by junction PCR. The efficiency of attB insertions in Rosa26–84–178 was 11.9% (5 out of 42) for group 1 and 6.8% (4 out of 59) for group 2. The embryo development rate of groups 1 and 2 (55.1%) is similar to the embryo development rate of the control group (63.1%), indicating that the prime editing system will not have a significant impact on embryonic development. (Figure 4, Table 10).

### 2.5. Integrated twinPE and SSR Introduced the Insertion of EGFP and Puro

**Figure 5:**
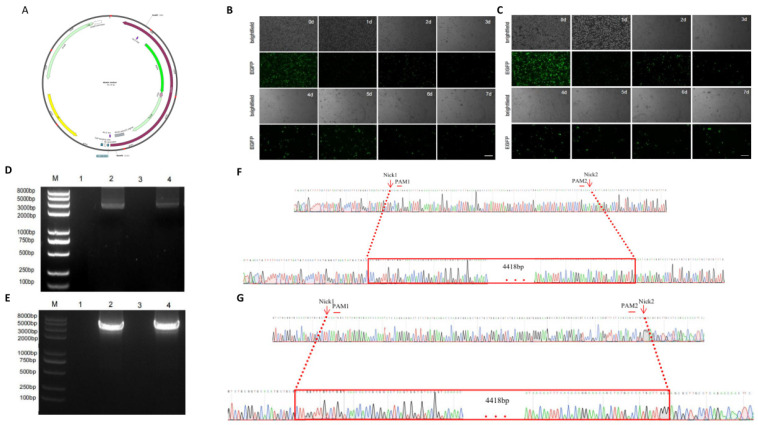
Insertion of *EGFP* and *Puro* at *FANCF* 456–591 and *CCR5* 3037–3126 in the genome of HEK293T cell. (**A**) Plasmid map of EGFP-Puro donor vector. (**B**) Positive cells that have accurate editions at *FANCF* 456–591 were screened from 0 to 7 d. (bar = 150 µm). (**C**) Positive cells that have accurate editions at *CCR5* 3037–3126 were screened from 0 to 7 d. (bar = 150 µm). (**D**) Junction PCR identification of *EGFP* and *Puro* insertion at *FANCF* 456-591. (**E**) Junction PCR identification of *EGFP* and *Puro* insertion at *CCR5* 3037–3126. (**F**) Comparison of Sanger sequencing between WT amplification fragments and *FANCF* 456–591 (*EGFP*-*Puro* ins) amplification fragments. (**G**) Comparison of Sanger sequencing between WT amplification fragments and *CCR5* 3037–3126 (*EGFP*-*Puro* ins) amplification fragments.

With the attB sequence inserted by twinPE and then using the BxbI site-specific recombinase and the donor vector containing the attP sequence and the target gene, the insertion of the target gene is possible by site-specific recombination. For the convenience of detection and screening, we selected *EGFP* (Enhanced Green Fluorescent protein) and *Puro* (Puromycin) as the target genes (total base number 4418 bp). (Figure 5A, Table 11).

In order to insert the large fragment of foreign gene at the inserted attB site, we amplified three fragments containing attP sequence, *EGFP*, *Puro*, and SV40polyA, ligated with pMD19T, and constructed the attP-*EGFP*-*Puro* donor vector. The motif2-integrated-twinPE expression vectors for *FANCF* 456–591 (attB ins) and *CCR5* 3037–3126 (attB ins), together with attP-*EGFP*-*Puro* donor vector and BxbI site-specific recombinase expression vector, respectively, were transfected by liposome into HEK293T cells. After 72 h, 1 mg/mL of puromycin was added, and puromycin concentration was maintained for 7 d to remove cells without expressing puro protein. (Figure 5B,C). After 7 d, we collected the surviving polyclonal cells, extracted the genome detected by junction PCR, and performed Sanger sequencing. The results show that integrated twinPE-mediated prime editing combined with site-specific recombination can effectively mediate exogenous gene insertion in large segments. (Figure 5D–G, Table 12).

## 3. Discussion

Prime editing enables high-efficiency deletion, substitution, or insertion of larger DNA sequences at targeted endogenous genomic sites in *human* cells. It provides more editing types than base editing (BE) and has similar editing precision and flexibility compared to CRISPR/Cas9 but with very low levels of off-target activities. However, considering the wide potential of the PE system for livestock gene breeding, it is necessary to improve its editing efficiency.

To address this issue, we developed two strategies to optimize the prime editor and analyzed its efficiency by inserting an attB sequence in the *goat* genome at target sites previously screened by the SSA reporter system. Of note, any exogenous gene can be integrated into this sequence by site-specific recombination.

In the first strategy, we improved the PE3 and twinPE system by incorporating RNA motif to the 3′ terminal of pegRNA and, as such, developed an integrated twinPE system. In fact, the RNA motif derived from the *HOXB13*-5′ UTR forms a stem-ring structure, which was previously shown to protect the RNA’s degradation [23]. Meanwhile, the spacer sequence and PBS sequence of pegRNA have complementary sequences. The pairing of the complementary stretches destroys the pegRNA single-strand structure and, as such, prevents complementary pairing with DNA. Importantly, the RNA motif maintains the structural independence of these two fragments.

We screened RNAmotif1 and RNAmotif2 from *HOXB13*-5′ UTR and added these sequences to the 3′ terminal of pegRNA. Next, we evidenced that RNAmotif2-pegRNA increased editing efficiency in HEK293T by 1.35-fold compared to WT pegRNA. Additionally, we evidenced that RNAmotif2-pegRNA provides notable enhancements over WT pegRNA at most target sites. For example, RNAmotif2-pegRNA-mediated *FANCF* 261 (6 bp del) achieved a 3.58-fold increased efficiency compared to WT pegRNA.

In the second strategy, we constructed all twinPE components—PE protein, pegRNA1, and pegRNA2—on the same plasmid. This plasmid provided an integrated twinPE system and included an *EGFP* detection marker for transfected cells. As such, this construct improved the transfection probability of the twinPE system into the same cell, with only one plasmid bearing all required components, and improved the detection efficiency of prime editing without the need for positive cell enrichment. The results indicated that the integrated twinPE editing efficiency was significantly improved and achieved 2.19-fold at *FANCF* 456–591 and 2.11-fold at *CCR5* 3027–3126. Based on these results, we speculated that integrated twinPE might have higher efficiency in hard-to-transfect cell lines.

To verify the effectiveness of the optimized PE system in integrating attB sequences into the *goat* genome, we used the SSA reporter system to select six pairs of target sites with higher cleavage efficiency of pegRNA-Cas9 and applied the enhanced RNAmotif2-integrated-twinPE system to insert an attB sequence into the *Rosa26* and *CCR5* gene loci of *goat* fibroblasts. In total, three out of six target sites could be edited by twinPE, while another three sites were not modified. In fact, an integration efficiency of 14.48% at *Rosa26*–80–183 demonstrated that the PE system works in *goat* cells with editing efficiencies that are directly related to the target site. Based on these results obtained in *goat* fibroblasts, we then inserted an attB sequence into *goat* parthenogenetic activation embryos genome following microinjection of the twinPE mRNA. With a concentration of 200 ng/µL PE2 mRNA, 50 ng/µL pegRNA1, and 50 ng/µL pegRNA2, editing was detected in 5 out of 42 embryos (11.9%) whereas with a concentration of 400 ng/µL PE2 mRNA, 100 ng/µL pegRNA1, and 100 ng/µL pegRNA2, editing could be detected in 4 out of 49 embryos, (6.8%). These results demonstrated that RNAmotif2-twinPE can mediate insertion in *goat* embryos. We chose parthenogenetically-activated *goat* embryos, as they are easily obtained and can indicate the impact of editing on embryonic development efficiency, similar to in vitro fertilization embryos and in vivo embryos. However, the limitation of parthenogenetically-activated *goat* embryos is that they cannot develop into a complete individual. Hence, in our future studies, we will insert attB sequences into the genomes of *goats* in vivo embryos by PE system and evaluate the developmental ability of transgenic embryos through embryo transfer. Of note, this must be based on the premise that the efficiency of prime editing can be significantly improved.

Finally, we verified whether the binding of twinPE and SSR can mediate the insertion of large exogenous genes into designated genomic loci. We decided to insert *EGFP* and *Puro* into *FANCF* and *CCR5* as this choice would enable direct observation and drug screening, while the length of this exogenous gene can meet the needs of transgenic animal production. We have proven that integrated twinPE with the SSR system can effectively mediate the insertion of exogenous genes in HEK293T cells. To evaluate the existence of the off-target integration at pseudo-attB sites, we compared sequences in the *human* genome that are similar to our attB sequence. The comparison results indicated that there are no pseudo-attB sites in the *human* genome similar to our sequence, indicating that our attB sequence is, in fact, highly specific. We subsequently conducted the same experiment in *goat* cells, but we could not select positive clones after screening. This may be due to the low transfection efficiency of primary cells and the low editing efficiency of prime editing in *goat* cells.

Overall, this study established a significantly improved prime editing system and evidenced its capacity to insert an attB sequence in the *goat* genome. This represents an important addition to the prime editing toolbox for future development and applications.

## 4. Materials and Methods

### 4.1. Plasmid Construction

The plasmids pCMV-PE2 (#132775), pGL3-U6-sgRNA-EGFP (#107721), and pSSA-1-3 (#35091) were obtained from Addgene. For expression of the integrated twinPE, each part of the plasmid backbone was amplified from pCMV-PE2 and pGL3-U6-sgRNA-EGFP using PrimeSTAR DNA Polymerase (Takara, Dalian, China) and linked by Gibson Assembly. For expression of the WT-PE2, the plasmid backbone was cut by NotІ-HF^®^v2 (NEB, Beijing, China) and KpnІ-HF^®^v2 (NEB, Beijing, China), after which each fragment was amplified from 2X_pX458_pSpCas9(BB)-2A-GFP (Plasmid #172221) and pCMV-PE2 and linked by Gibson Assembly. The spacer oligos, scaffold oligos, 3′ extension oligos of pegRNA, and RNA motif-3′ extension oligos of RNA motif-pegRNA were synthesized, annealed, and assembled by DNA ligase (Takara, Dalian, China).

### 4.2. Cell Culture and Transfection

HEK293T and *goat* fibroblast cells isolated from the ear margin of female *goats* aged 1 to 3 months were grown in Dulbecco’s modified Eagle’s medium (DMEM, Gibco, New York, NY, USA) or DMEM/F-12 (Gibco, New York, NY, USA), respectively, supplemented with 10% fetal bovine serum (FBS, Gibco, Queensland, Australia) at 37 °C in humidified incubator with 5% CO_2_. After seeding cells onto a 12-well plate 1 day before transfection, each well was transfected with 2 µg of plasmids and 10 µL PEI (Polysciences, Shanghai, China) at approximately 60% cell confluency. For the PE3 system, the proportion of plasmids was PE2/pegRNA/sgRNA = 9/3/1. For the twinPE system, the proportion of plasmids was PE2/pegRNA1/pegRNA2 = 6/1/1. For the SSA system, the proportion of plasmids was pSSA-1-3/PE2(WT)/sgRNA/pRL-SV40 = 18/15/4/2. One hour before transfection, the medium was changed to an FBS-free medium and to DMEM supplemented with 10% FBS twelve hours after transfection.

### 4.3. SSA Reporter System

HEK293T cells were seeded onto a 24-well plate and, the next day, transfected with pSSA-1-3 plasmids of each target site with pCMV-WT-PE2, sgRNA, and pRL-SV40 in triplicate. Forty-eight hours after transfection, cells were washed with PBS and lysed with 1×cell lysis buffer of TransDetect^®^ Double-Luciferase Reporter Assay Kit (Transgene, Beijing, China). Following centrifugation at 12,000× *g* for 20 min at 4 °C, 20 µL supernatant was added into 3 wells of a 96-well plate. Then, 50 µL Luciferase Reaction Reagent was added into each well, the plate was mixed by vibration, and the chemiluminescence of firefly luciferase was detected with a luminometer. Next, 50 µL Luciferase Reaction Reagent ІІ was added into each well, the plate was mixed by vibration, and the chemiluminescence of renilla luciferase was detected. The relative cutting efficiency of pegRNA was reflected by the ratio of firefly and renilla luciferase.

### 4.4. Genome DNA Preparation and Detected with PCR

Three days after transfection, cells were washed with PBS and lysed with trypsin (Gibco, NY, USA), after which genomic DNA was extracted with a Genomic DNA kit (Tiangen, Beijing, China). Genomic DNA was amplified with PCR primers and revealed with agarose gel electrophoresis. For junction PCR, edited cells which were revealed objective stripe while WT cells were not. The objective stripe was purified and cloned in pMD19T for subsequent Sanger sequencing.

### 4.5. Targeted Deep Sequencing and Data Analysis

Sequences ranging from 250 to 300 bp, including the editing, were amplified by PCR and sequenced on an Illumina MiSeq. Alignment of amplicon sequences to a reference sequence was performed using CRISPResso2 (Cambridge, MA, USA). CRISPResso2 was run in BE mode for single base mutation and in HDR mode for insertion or deletion edits.

### 4.6. Limited Dilution Method for Screening Monoclonal Cells

Three days following transfection, cells were washed with PBS and digested with trypsin. Following centrifugation at 1050 rpm for 5 min, it was resuspended in DMEM/F-12 medium at 1/10,000 dilution in a 10-cm culture dish. After 7 days of cultivation, the culture medium was discarded, washed with PBS, and digested with trypsin. The single-cell clones were gently aspirated with a 100 μL pipette. Then, a portion of the cells was extracted for identification, while the remaining part was used to continue cultivation.

### 4.7. Puromycin Method for Screening Polyclonal Cells

Three days following transfection, the cell culture medium was supplemented with 1 mg/mL of puromycin to remove cells not expressing puro protein. The cell culture medium was replaced every 2 d, and the puromycin concentration was maintained for 7 d. The positive cell clusters generated in the cell culture dishes could express green fluorescent protein.

### 4.8. Collection and In Vitro Culture of Goat Oocytes

Upon collection, *goat* ovaries were transported to the laboratory in physiological saline containing penicillin and streptomycin. The connective tissue around the ovaries was removed to fully expose the follicles. After rinsing the ovaries with 75% alcohol and physiological saline, they were placed in a 60-mm cell culture dish with PBS. Then, the follicles were punctured on the surface of the ovaries to allow the cumulus-oocyte complexes (COCs) to flow into the PBS fluid along with the follicular fluid. COCs with uniform cytoplasm, intact morphology, and complete cumulus cell coating were selected by using an oocyte probe under a stereomicroscope. The COCs were washed twice in PBS, transferred to an oocyte maturation medium (KSOM, Sigma, Saint Louis, MO, USA), and cultured in a cell culture incubator at 38.5 °C in a humidified incubator with 5% CO_2_.

### 4.9. Parthenogenetic Activation of Goat Oocytes

Following 22 h in vitro culture, the granulosa cells on the surface of the oocytes were removed, after which the oocytes were transferred to 0.1% hyaluronidase (HY, Sigma, Saint Louis, MO, USA) for 5 min to remove any remaining granulosa cells. Next, oocytes with the first polar body and intact form were selected and transferred to ION ionomycin (Sigma, Saint Louis, MO, USA) droplets and blown and aspirated in two ION ionomycin droplets for 3–5 min in sequence. After 5 min restoration in KSOM, the oocytes were sequentially washed in two 6-DMAP (Sigma, Saint Louis, MO, USA) microdroplets, collected in the third 6-DMAP droplet, and cultured in a cell culture incubator at 38.5 °C in a humidified incubator with 5% CO_2_ for 4 h.

### 4.10. Microinjection of Goat Embryos

Four microdroplets of microinjection solution were formed in the cover of a 35-mm culture dish, covered with mineral oil (Sigma, Saint Louis, MO, USA), and preheated to 37 °C. Then, the mRNA of twinPE system was mixed and centrifuged at 10,000 rpm/min for 20 min at 4 °C. Each embryo was injected with 7 pL RNA, washed in G1 embryo culture medium (Thermo Scientific, Boston, MA, USA) droplets, and transferred to G1 embryo culture medium in a four-well plate.

Then, 400 parthenogenetic activation embryos were divided into two groups. Group 1 was microinjected with 200 ng/µL PE2 mRNA, 50 ng/µL pegRNA1, and 50 ng/µL pegRNA2 and group 2 was microinjected with 400 ng/µL PE2 mRNA, 100 ng/µL pegRNA1, and 100 ng/µL pegRNA2. After 5 days of cultivation at 38.5 °C in a humidified incubator with 5% CO_2_, 42 morulas of group 1 and 59 morulas of group 2 were collected, each embryo was cracked individually, and twinPE efficiency was detected with junction PCR.

### 4.11. Statistical Analysis

All statistical analyses were performed using GraphPad Prism 6.0. Data were presented as standard error of the mean (SEM) and analyzed with a paired *T*-test from three independent experiments. Differences were considered statistically significant at *p* < 0.05.

## 5. Conclusions

In this paper, we describe two strategies for developing the prime editor. Adding a *HOXB13*-5’ UTR RNA motif to the 3’ terminal region of the pegRNA or integration of all the twinPE components into one plasmid enhanced PE editing efficiency by 3.58-fold and 2.19-fold, respectively. Following the screening of efficient target sites in *Rosa26* and *CCR5* within the *goat* genome and the insertion of an attB sequence into these sites and by verifying the feasibility and efficiency of microinjection-mediated insertion of the attB sequence into *goat* parthenogenetic activation embryos, we demonstrated the applicability of PE in *goat* transgenic breeding to insert large fragments of foreign genes into the attB site by combining twinPE with SSR.

## Figures and Tables

**Table 1:** Sequences of *HOXB13*-5′ UTR RNAmotif1 and RNAmotif2.

Name	Sequence
RNAmotif1	GCATGTGGGATCTAGCTCCCTGACCAGGGATCGAACCTGGGCCCC
RNAmotif2	CTTAGCCACTGCACCAGGGAATTCCCTGCCAGCGATTT

**Table 2:** pegRNA sequences in HEK293T cells.

pegRNA	Spacer	3′ Extension
*FANCF* 594 (6 bp ins)	GTCTGAGGCAAGCGCTCCCAC	AGCCTGTGGGATCCGGAGCGCTTGCCT
*CCR5* 3037 (89–38 bp sub)	GTCCACTATGCTGCCGCCCAG	GGCTTGTCGACGACGGCGGTCTCCGTCGTCAGGATCATGGCGGCAGCAT
*CCR5* 3126 (89–38 bp sub)	GGTACCTATCGATTGTCAGG	ATGATCCTGACGACGGAGACCGCCGTCGTCGACAAGCCGACAATCGA
*FANCF* 179 (A to T)	GTCGGCATGGCCCCATTCGCA	TCCAGAGCCGAGCGAATGGGGCCAT
*FANCF* 261 (6 bp del)	GTCCGGGATTAGCGAACTTCC	GACGTCACAGTGACCGAGGGAGTTCGCTAATCC
*FANCF* 327 (6 bp sub)	GCTGGAGAACCGGGCCCTCG	GCTGCACCAGGTGGTAACGAGGGATCCCCCCGAGGGCCCGG
*FANCF* 456 (135–38 bp sub)	GTGCACATGCTGCGCTTCAA	GGCTTGTCGACGACGGCGGTCTCCGTCGTCAGGATCATAAGCGCAGCATGT
*FANCF* 591 (135–38 bp sub)	GTCTGAGGCAAGCGCTCCCAC	ATGATCCTGACGACGGAGACCGCCGTCGTCGACAAGCCGGAGCGCTTGCCT
*CCR5* 2317 (6 bp ins)	GCTTGAGCCCGGGATGGTCC	ATGGCTCACTGCAGCCTGGAGGATCCCCATCCCGG
*CCR5* 3272 (6 bp del)	GTATGGAAAATGAGAGCTGC	ATCATCTTTACCAGATCTCAAGGTCTTCATTACACCTGCAGCTCTCATTTTCC
*CCR5* 4848 (6 bp sub)	GTGGATTTGGGTTGGAAGTGA	TTCTCTCTGACTCCTCTCTGGGATCCACTTCCAACCCAAAT

**Table 3:** Primers for identification of editing by junction PCR.

Target Site	Primer For	Primer Rev
*FANCF* 594 (6 bp ins)	GGCAAGCGCTCCGGATCC	TGGTGCAGCAACTCTTTC
*CCR5* 3037–3126 (89–38 bp sub)	ATGATCCTGACGACGGAG	GGTGGAACAAGATGGATTATCA
*FANCF* 456–591 (135–38 bp sub)	ATGATCCTGACGACGGAG	GCTAGTCCACTGGCTTCT
*FANCF* 327 (6 bp sub)	ACCAGGTGGTAACGAGGGATCC	GTCTCAAGCACTACCTACG
*CCR5* 2317 (6 bp ins)	TTGAGCCCGGGATGGGGATCC	ATTGCCTACATAGATGTCTAC
*CCR5* 4848 (6 bp sub)	ATTTGGGTTGGAAGTGGATCC	GAAGTCAGAAGCATTCAGTG

**Table 4:** Primers for identification of editing by deep targeted sequencing.

Target Site	Primer For	Primer Rev
*FANCF* 594 (6 bp ins)	CACCGCTATCACCTTCAG	CGCTTCAATGGCTATAGAGAGA
*FANCF* 327 (6 bp sub)	AACCAGTGGAGGCAAGAG	GGTCTTCATCAGAGAGTCCT
*FANCF* 179 (A to T)	GTCTCAAGCACTACCTACG	GCCTGGAAGTTCGCTAAT
*FANCF* 261 (6 bp del)	GTCTCAAGCACTACCTACG	TGGAGTGTCTCCTCATCG
*FANCF* 456–591 (135–38 bp sub)	ATGAGGAGACACTCCAAGA	CACCGCTATCACCTTCAG
*CCR5* 2317 (6 bp ins)	ATTAGCTTGGTGTGGTGG	GTTGTTGTTGTTGTTGTGAG
*CCR5* 4848 (6 bp sub)	GGAGGTATTCGTAAGGATGG	GAAGTCAGAAGCATTCAGTG
*CCR5* 3272 (6 bp del)	CTGTGCCTCTTCTTCTCATT	CTCTGGAATCTTCTTCATCATC
*CCR5* 3037–3126 (89–38 bp sub)	ACATCTACCTGCTCAACCT	ATCACACTTGTCACCACC

**Table 5:** Enzyme digestion system of integrated twinPE backbone.

Component	Volume
2000 ng integrated twinPE plasmid	X µL
BsaI-HF^®^v2(NEB)	1 µL
BbsI-HF^®^v2(NEB)	1 µL
Buffer	3 µL
ddH_2_O	To 30 µL

**Table 6:** Modifications of each part of integrated twinPE.

Component	Modification
Scaffold top	5′ phosphorylated.
Scaffold bottom	5′ phosphorylated.
Spacer 1 top	5′ accg overhang, 3′ gtttt overhang
Spacer 1 bottom	5′ ctctaaaac overhang
Spacer 2 top	5′ caccoverhang, 3′ gtttt overhang
Spacer 2 bottom	5′ caaaatctc overhang
Extension 1 top	5′ gtgc overhang
Extension 1 bottom	5′ taaa overhang
Extension 2 top	5′ gtgcoverhang
Extension 2 bottom	5′ aaaaoverhang

**Table 7:** Predicting efficiency and sequence of pegRNA at *CCR5* and *Rosa26* loci in *goat* genome.

pegRNA	Direct	Predicting Efficiency	SSA	Spacer	3′extension
*Rosa26* 80	F	15.07	6.03	GAGGTCGTCTGGCCGGTATCT	GGCTTGTCGACGACGGCGGTCTCCGTCGTCAGGATCATTCTGGGGGTC
*Rosa26* 183	R	13.68	5.51	GCTCCCTCTTCGGTTGCCGCA	ATGATCCTGACGACGGAGACCGCCGTCGTCGACAAGCCGGCAACCGAA
*Rosa26* 233	F	7.17	8.94	GGAAAAGGCTCGAATCCGGA	GGCTTGTCGACGACGGCGGTCTCCGTCGTCAGGATCATGGATTCGAGC
*Rosa26* 372	R	14.22	7.52	GTAGGCGGCTGCGCACTCCCG	ATGATCCTGACGACGGAGACCGCCGTCGTCGACAAGCCGAGTGCGCAG
*Rosa26* 581	F	10.22	9.33	GCGGTTTACCCGCCGCCTGCT	GGCTTGTCGACGACGGCGGTCTCCGTCGTCAGGATCATAGGCGGCGGG
*Rosa26* 633	R	8.31	7.74	GCGGCCCCTTGTTATTGGCTC	ATGATCCTGACGACGGAGACCGCCGTCGTCGACAAGCCCCAATAACAA
*Rosa26* 638	F	12.14	8.22	GACGGCCCCTTGTTATTGGCT	GGCTTGTCGACGACGGCGGTCTCCGTCGTCAGGATCATCAATAACAAG
*Rosa26* 740	R	14.67	3.49	GTGCCACGTTGCGCAGGGGCG	ATGATCCTGACGACGGAGACCGCCGTCGTCGACAAGCCCCCTGCGCAA
*Rosa26* 1017	F	17.4	2.17	GAAGGCCGCACCCTTCTCAGC	GGCTTGTCGACGACGGCGGTCTCCGTCGTCAGGATCATGAGAAGGGTG
*Rosa26* 1124	R	6.57	2.78	GACATGCGATGACGAGATCGC	ATGATCCTGACGACGGAGACCGCCGTCGTCGACAAGCCATCTCGTCAT
*Rosa26* 1160	F	10.13	1.91	GAGTCTCTCCTCGATTATGGG	GGCTTGTCGACGACGGCGGTCTCCGTCGTCAGGATCATATAATCGAGG
*Rosa26* 1212	R	13.29	2.34	GATACCTGCTCGATCCACTGC	ATGATCCTGACGACGGAGACCGCCGTCGTCGACAAGCCGTGGATCGAG
*CCR5* 902	F	4.80	6.62	GGGAATCGGGACTTGACAAC	GGCTTGTCGACGACGGCGGTCTCCGTCGTCAGGATCATGTCAAGTCCC
*CCR5* 996	R	8.88	2.98	GTTGGTGAGAGAACTACAAGC	ATGATCCTGACGACGGAGACCGCCGTCGTCGACAAGCCTGTAGTTCTC
*CCR5* 1022	F	8.04	0.83	GCTCACCAAGGAGTGAAAGAC	GGCTTGTCGACGACGGCGGTCTCCGTCGTCAGGATCATTTTCACTCCT
*CCR5* 1091	R	11.09	4.64	GAACTTCCCCAAAGACCCACA	ATGATCCTGACGACGGAGACCGCCGTCGTCGACAAGCCGGGTCTTTGG
*CCR5* 1301	F	7.47	2.98	GCTATGTGTAAGCTAACTCC	GGCTTGTCGACGACGGCGGTCTCCGTCGTCAGGATCATGTTAGCTTAC
*CCR5* 1441	R	4.62	2.62	GCTAACACTCTGTTTACTAGA	ATGATCCTGACGACGGAGACCGCCGTCGTCGACAAGCCAGTAAACAGA
*CCR5* 2671	F	10.64	0.15	GATAACTGCAGTGACTCTAAC	GGCTTGTCGACGACGGCGGTCTCCGTCGTCAGGATCATAGAGTCACTG
*CCR5* 2753	R	6.22	1.23	GTCGGAACTTCTCCCCCACGA	ATGATCCTGACGACGGAGACCGCCGTCGTCGACAAGCCTGGGGGAGAA
*CCR5* 2752	F	4.95	1.41	GCCATCATCTATGCCTTCGTG	GGCTTGTCGACGACGGCGGTCTCCGTCGTCAGGATCAT
*CCR5* 2810	R	7.18	3.26	GACAGCCTTTGCAGAAGCGGC	ATGATCCTGACGACGGAGACCGCCGTCGTCGACAAGCCGCTTCTGCAA
*CCR5* 3535	F	6.45	2.15	GGCACTGCACAGGCAAAACT	GGCTTGTCGACGACGGCGGTCTCCGTCGTCAGGATCATTTTGCCTGTG
*CCR5* 3657	R	13.85	1.35	GCTTTCTGCAACATTGGCCA	ATGATCCTGACGACGGAGACCGCCGTCGTCGACAAGCCCCAATGTTGC

**Table 8:** Primers for identification of attB insertion into *goat* genome by junction PCR.

Target Site	Primer For	Primer Rev
*CCR5*–902–996	ATGATCCTGACGACGGAGAC	GGCTTGCTGGTGATAATGAA
*CCR5*–1301–1441	ATGATCCTGACGACGGAGAC	GAATGTCCTTAGCAGTCCATC
*CCR5*–2752–2810	ATGATCCTGACGACGGAGAC	GCGGTGAGAAGATTGCTATT
*Rosa26*–80–173	ATGATCCTGACGACGGAGAC	CCGAGGCTGTAACTGACA
*Rosa26*–233–372	ATGATCCTGACGACGGAGAC	TGAGCTTCAGCAGCCAATC
*Rosa26*–633–740	ATGATCCTGACGACGGAGAC	GAACTCCCATAAAGGTATTGC

**Table 9:** Primers for identification of attB insertion into *goat* genome by deep target sequencing.

Target Site	Primer For	Primer Rev
*CCR5*–902–996	AATGTGAGAATGGGAATC	CTTTCACTCCTTGGTGAG
*CCR5*–1301–1441	CCCTAATGATGCTATGTG	GCCTGAGCGACTAACACT
*CCR5*–2752–2810	TGCATCAACCCCATCATC	TGGAAGACTGGACAGCCT
*Rosa26*–80–173	ACCTAGAGAAGAGGCTGTG	CCGAGGCTGTAACTGACA
*Rosa26*–233–372	AGAAGGGAGCGGAAAAGG	TGCGAGAGTCCCTAGGCG
*Rosa26*–633–740	AGGGAGGGTCAGTGAAAG	TAGTCTTGTCCTTCAGGCA

**Table 10:** Single embryo editing efficiency of attB insertion at *goat Rosa26*.

Group	Embryo Development Rate	Edit Positive Rate
1	55.1% (101/183)	11.9% (5/42)
2		6.8% (4/59)
Control	63.1% (53/84)	0% (0/53)

**Table 11:** Templates and primers for the amplification of EGFP-puro donor plasmid.

Fragment	Template	Primer For	Primer Rev
EcoRI–attP–SA–EGFP–kpnI	pGL3–U6–sgRNA–EGFP	①agagctagaaatagcaagttaaaataaggctagtccgttat caacttgaaaaagtggcaccgagtcg ②ccggaattccggggtttgtctggtcaaccaccgcggactcag tggtgtacggtacaaaccccttgcattctcag	cggggtaccccgcttgtacagctcg
kpnI–P2A–Puro–NHeI	pMXs–IRES–Puro	cggggtaccccggccacgaacttctctctgttaaagcaagcaggagacgtggaagaaaaccccggtcctatgaccgagtac	cgcggatccgcgctagctagctagtcaggcaccgggct
NHeI–SV40polyA–BamHI	pGL3–U6–sgRNA–EGFP	ctagctagctagagcggccgcgactctaga	cgcggatccgcgtaagatacattgatga

**Table 12:** Primers for identification of *EGFP* and *Puro* insertion by junction PCR.

Target Site	Primer For	Primer Rev
*FANCF* 456–591 (*EGFP*–*Puro* ins) For	CCTTGCATTCTCAGAGTCAG	CCTGCGCTTTACAGGTCTCC
*FANCF* 456–591 (*EGFP*–*Puro* ins) Rev	AGCACTACCTACGTCAGCAC	TAAGATACATTGATGAGTTT
*CCR5* 3037–3126 (*EGFP*–*Puro* ins) For	CCTTGCATTCTCAGAGTCAG	TCACCTGCATAGCTTGGTCCA
*CCR5* 3037–3126 (*EGFP*–*Puro* ins) Rev	AACTTCATTGCTTGGCCAAAAA	TAAGATACATTGATGAGTTT

## Data Availability

All data generated or analyzed during this study are included in this published article.
